# Virtual Obstacles for Sensors Incapacitation in Robot Navigation: A Systematic Review of 2D Path Planning

**DOI:** 10.3390/s22186943

**Published:** 2022-09-14

**Authors:** Thabang Ngwenya, Michael Ayomoh, Sarma Yadavalli

**Affiliations:** Department of Industrial and Systems Engineering, University of Pretoria, Hatfield, Pretoria 0028, South Africa

**Keywords:** mobile robot navigation, virtual obstacles, sensor incapacitation, environmental conditions

## Abstract

The field of mobile robot (MR) navigation with obstacle avoidance has largely focused on real, physical obstacles as the sole external causative agent for navigation impediment. This paper has explored the possible option of virtual obstacles (VOs) dominance in robot navigation impediment in certain navigation environments as a MR move from one point in the workspace to a desired target point. The systematically explored literature presented reviews mostly between the years 2000 and 2021; however, some outlier reviews from earlier years were also covered. An exploratory review approach was deployed to itemise and discuss different navigation environments and how VOs can impact the efficacy of both algorithms and sensors on a robotic vehicle. The associated limitations and the specific problem types addressed in the different literature sources were highlighted including whether or not a VO was considered in the path planning simulation or experiment. The discussion and conclusive sections further recommended some solutions as a measure towards addressing sensor performance incapacitation in a robot vehicle navigation problem.

## 1. Introduction

Over the years, mobile robots (MRs) have been deployed to smartly assist humans in routines requiring navigation intelligence in the work environment [1,2]. This has partly been facilitated through the use of sensors. Sensor technology types coupled with guidance, control, and navigation decision-making algorithms are primarily responsible for the intelligence in MR path planning. The appearance of intelligence associated with MRs is capable of failing when they are exposed to certain environmental conditions capable of causing sensor malfunction. A few of these malfunctions can be seen in extreme weather conditions such as overheating temperatures with extreme heat emissions (e.g., emissions from groundwater in mines and gas leaks underground in the case of intelligent underground mine rovers) [3] and in clustered domains such as collapsed buildings, cave-ins, or fire outbreak in buildings in the case of search and rescue robots, among other scenarios. According to [3], excessive heat, wind, and other obstacles can hinder the functional ability of the sensors hence, causing MRs to react abruptly. The literature has also confirmed that an inertial measurement sensor is prone to failure when there is an electromagnetic interference with its signal emissions [4]. The malfunctioning of a sensing device often results in the display of false data, hence impacting the overall accuracy and behavioural intelligence of a MR [5]. Another shortcoming associated with sensors in MRs is the little scope of significant distance estimation and blind areas [6] which can be orchestrated in the environmental domain. In the earlier review exercises [7,8,9,10] on 2D robot navigation (RN), efficacy was mostly measured and assessed based on algorithmic strength.

As a result, the current review is focused on investigating RN incapacitation based on environmental conditions that can impede the performance of sensors. The review is anchored on the fact that sensory incapacitation, as with algorithmic ineffectiveness, can hinder a robot from successfully navigating to a desired target point (TP). Sensory incapacitators in the context of this review are not visible, physical objects, but rather invisible or unseen, virtual phenomena as earlier discussed. Potential sensory incapacitation environments for robotic vehicles are often facilitated by magnetic fields, electric fields, clustered and dark environments, environments infiltrated with nuclear radiations and harmful gases, among others. Sensors that are negatively impacted in these environments include but are not limited to LiDAR, ultrasonic, radar, GPS, and infrared sensors [11]. In underground mining for instance, poor conditions such as dangle nano-size dust particles and unclear lighting can significantly limit the performance of a vision sensor [12]. Sensory incapacitation herein is not about mechanical or electrical fault on a mounted sensor, but rather the impact of invisible, unseen, external environmental influences. These invisible sensory incapacitating phenomena are referred to as virtual obstacles (VOs) in the context of this research. VOs are neither visible to the human eyes nor the mounted sensory devices on a MR; rather, they remain invisible and can affect the navigation of a robot towards its desired TP by interfering with the transductive effectiveness of the sensor, hence resulting in wrong metric output. There is a gap in exploring challenges associated with functionality of sensors when MRs are deployed in environments containing VOs.

The review exercise herein is aimed at systematically analysing research works in the field of 2D MR navigation with a view towards exploring how much attention was attributed to understanding VOs as possible causes of sensing incapacitation which can result in poor path planning (PP) as much as an ineffective algorithm. The review aims to understand sensors, sensing incapacitation domains, and how these can influence 2D domain navigating robot in navigation environments such as the underground domains for mining activities, cluttered domains, harmful gaseous environments, and others. The rest of the paper is divided into two additional sections, viz., Section 2, which addresses a review of specific research works and their algorithmic and sensing incapacitation considerations for effectiveness in robot PP, and Section 3, which focuses on discussions, findings, recommendations, and future work.

## 2. Most Commonly Used Navigation Methodologies for MRs

Researchers have applied several methodologies in addressing the 2D RN problem amidst workspace obstacles with considerations given mostly to validating the algorithmic efficacy or inefficacy in the deployed workspaces. In these research works, some papers only discussed the use of an algorithm without a mention of hardware utilisation, especially when the validation process is simulation based. This review has focused on the most commonly used classical and heuristic approaches in 2D robot path planning in both algorithmic control and sensory incapacitation discussions regarding the efficacy or inefficacy of PP in diverse navigation environments. In the reviewed papers [13,14,15,16,17,18,19,20,21,22,23,24,25,26,27,28,29,30,31,32,33,34,35,36,37,38,39,40,41,42,43,44,45,46,47,48,49,50,51,52,53,54,55,56,57,58,59,60,61,62,63,64,65,66,67,68,69,70,71,72,73,74,75,76,77,78,79,80,81,82,83,84,85,86,87,88,89,90,91,92,93], a noticeable gap can be observed in the literature regarding information and discussions about the likely environments where sensors may fail in the case of experimental validations which can lead to the malfunctioning or inefficacy in the algorithmic outputs based on environmental influences on the mounted sensors. The researchers very often, present discussions on the efficiency of the deployed algorithms without referring to sensory incapacitation even in a possible medium to high-risk experimental environment. Mostly, these algorithms were validated on simulation platforms, with a few validated experimentally using real dynamic obstacles (DOs) and real static obstacles (SOs) environment, with a mention of VOs which can act as sensor incapacitators.

### 2.1. An Overview of Algorithms and Navigation Approaches

The following Section 2.1.1 and Section 2.1.2 have presented a review of various path planning research works in a bid to investigate if any of these algorithms were deployed to address possible VOs as much as the real obstacles in a robot navigation workspace. In addition, a mild classification was carried out to be sure which algorithms were deployed in a strictly simulation environment, an experimental environment, or a hybrid environment. Very often, researchers in this problem domain are mostly concerned about the efficacy of their algorithms in the presence of physical workspace obstacles. Hence, this section is not seeking to compare the efficacy of algorithms or their degree of sensorial independence in a robot navigation mission (i.e., if an algorithm can be used independent of a sensor or not).

#### 2.1.1. Classical Approaches

The following classical techniques have been reviewed in this section: Simultaneous Localisation and Mapping (SLAM), as well as some commonly used algorithmic solutions that can either be fused into the concept of SLAM to facilitate its localization and mapping features or be independently deployed and used directly as standalone control algorithms in the navigation and control of 2D robotic vehicles. These include Light Detection and Ranging (LiDAR), Vector Field Histogram (VFH) and the Artificial Potential Field (APF)/Virtual Force Field (VFF).

##### Simultaneous Localisation and Mapping (SLAM)

SLAM is one of the most used methodologies that addresses the path planning problem via the construction of a workplace map with no prior knowledge of the environment by a navigating robot. It further localises the MR within the map without any human involvement, as discussed by Taheri and Xia [13]. It was further asserted by Taheri and Xia [13] that using low-level sensors makes utilising SLAM technique difficult. As a result, SLAM is associated with observation errors associated with sensors and caused by the changes in physical factors of the environment. Moreover, other researchers [14,15,16,17,18,19,20,21] conducted research works on SLAM for RN with real obstacles; however, there was no mention of VOs in their research. A need to investigate and explore possible navigation inefficiencies or inaccuracies from the point of view of sensor impairment cannot be overemphasised. In [12], the author implemented SLAM in underground mining and found that the challenges were directly linked to VOs such as dust and illumination challenges for the sensors. However, there is less literature that addresses SLAM efficiency where the different types of sensors are capable of malfunctioning due to VOs orchestrated by environmental influences. Despite SLAM being an effective path planning technique, the thinking of the future in experimental path planning is in both algorithmic and sensory assessment. For instance, will an incorrect localisation of the navigating robot always be linked to algorithmic deficiency? Can there be some temporary or permanent perceptory conditions resulting from the environment hence contributing to the poor signal prompting and incorrect readings? The same thinking applies to mapping. Could it be that some areas within the workspace are not accessible by the sensors due to unseen influences? Inaccuracies resulting from virtual conditions can be a source of navigation blind spots and are seen as posing critical challenges to a navigating robot especially when all attention is on the physical obstacles and visual environment.

##### Light Detection and Ranging (LiDAR)

LiDAR is an eminent dynamic distance detecting path planning sensor system which is utilised as a range estimation sensor which consistently sends a beam of light and utilises pivoting radiations at a steady rate. It also registers the distance between the object and itself with high precision. LiDAR improves outcomes when combined with different sensors [22]. In [12], the author highlighted that vision sensors are greatly restricted by VOs, which may affect the success of LiDAR beams in the underground mines. Over the years, researchers have explored this technique to make more useful improvements. Ghorpade et al. [23] proposed an efficient OA model using the 2D LiDAR for an MR to accomplish proper constant execution and improve the precision of OA focused on independent mechanical frameworks intended for military applications. However, the paper does not address the limitations that the sensors used have in this environment.

Madhavan and Adharsh [24] used a deliberate methodology to dodge impediments on most minor expense work guidelines, which are limited to simulation environment with static obstacles. Additionally, Baras et al. [25] used LiDAR and Raspberry Pi to address navigation problems while the autonomous vehicle avoided impediments. The results show that the approach can navigate safely in less luminous environments. Future adjustments might anticipate impediments to movement and may more efficiently explore in a dynamic workspace. Similarly, Dong et al. [26] and Ren Yee et al. [27] used real-time experiments in the presence of static and dynamic obstacles. However, it could be assumed that the experiments were conducted in a workspace that is void of VOs capable of resulting in the failure of the mounted sensors, as the algorithm was effective without failing. However, as a remote sensing device that uses laser pulse-like lighting beams for high resolution maps in surveillance, among other uses, deploying the LiDAR system in, for instance, an underground facility for autonomous path planning in a mining environment may result in visibility related challenges. LiDAR sensors facilitate robotic vehicles to visualise the ambient environment by generating and measuring several data points, and then creating a dynamic navigation map of the static or changing environment. The LiDAR sensor has a deficiency in measuring distances through interceptions such as heavy rain, snow, and fog. In addition, the LiDAR sensor measurement capability can be adversely impacted by contamination from sunlight during the day as pointed out in Atmospheric Chemistry and Physics, European Geosciences Union [28]. When LiDAR receives scattered radiations from the Sun, they easily become saturated, following that the solar radiation has so much influence on a diverse set of wavelengths. In general, the performance of LiDAR depreciates as the weather conditions deteriorates. How all of these culminate into a negative impact on a robot and to what degree remains an open investigation to be carried out.

##### Vector Field Histogram (VFH)

The literature from [29,30,31,32,33,34,35,36] highlight that there is a gap in applying this technique in environments with VOs, because even in the real world it is not considered that experiments involving sensors can malfunction and cause wrong algorithmic outputs. The VFH was pioneered by Borenstein and Koren [29]. The technique was very robust and efficient. Ulrich and Borenstein improved the VFH in 1998 [30] and 2000 [31]. The technique was developed to diminish the restriction of potential-field strategies (i.e., robot motions while dodging the obstacles) [32]. Yim and Park [33] used VFH in RN with SOs. Kumar and Kaleeswari [34] implemented the VFH in a robot with DOs and SOs. Future work will consider the use of a potential field strategy. Alagic et al. [35] proposed a modified VFH technique in an MR framework. Their VFH calculation gave local movement arranging and obstruction evasion dependent on ready sensor estimations. Results demonstrated the VFH calculation’s capability to explore RN prior to the TP evading impediments. The disadvantage with this technique is that it gives mediocre results for local PP regarding travel time and distance covered. Diaz and Marin [36] improved on the algorithm proposed by [30].

##### Artificial Potential Field (APF)/Virtual Force Field (VFF)

In [37,38,39,40,41,42,43,44,45,46,47,48,49,50,51], the application of the VFF technique even when mixed with other approaches was limited to user-friendly environments. Hence, there is still insufficient literature where the efficiency and effectiveness of this approach is tested in the presence of VOs. The APF PP innovation previously proposed by Khatib is on a fundamental level appropriate for constant control [37]. APF is also known as VFF, which Borenstein and Koren [38] pioneered. The drawback is that it falls into the local minima trap (LMT) and neglects the TP. The essential thought of APF is to make the robot move using forces such that obstacles produce a repulsive force (RF), and TP delivers an attractive force (AF) on a robot. The paper [39] provides crucial functions in understanding APF. Chiang et al. [40] used APF-stochastic reachable strategy for PP in complex workplaces. Extended work by Malone et al. [41] for PP was in a highly intricate and dynamic workplace with impediments. Sudhakara et al. [42] investigated OA and navigation of a wheeled robot using amended APF in unstructured environments. Results showed that the enhanced APF may be adequately used in the direction arranging of wheeled robots and can be applied progressively in real-time situations. The improved APF calculation adjusts well in specific and complex conditions with a short travel time. Lu et al. [43] and Lin et al. [44] algorithm dependent on the improved APF to tackle the issue of local optimum.

A discrete artificial potential field (DAPF) for robot PP introduced by Lazarowska [45] utilises the idea of an APF and alters it for use in a discrete setup space. Results showed that the DAPF calculation is fit for finding a crash-free way for a robot in dynamic and static conditions. The advantage of this is the close ongoing activity, which makes it helpful for practical applications. Moreover, a new pattern in RN research is the use of a hybrid approach (HA) to accomplish better outcomes. Shin and Kim [46] pioneered a HA that combines positioning risk (PR) and the APF. They designed a flowchart that mapped out the methodology premised on the use of a temporary goal (TG). e The algorithm is triggered when the MR does not reach its TP because of LMT caused by obstacles. Results from their paper showed that the proposed PR-APF generated more than 90% success paths while the APF failed to generate up to 50% success paths which constitutes a significant limitation for the unenhanced APF method. Another HA is the hybrid virtual force field (HVFF) approach. This approach integrates the virtual force field (VFF), virtual obstacle concept (VOC), and the virtual goal concept (VGC). The HVFF flowchart as presented in their paper showed a few navigations rules. One of these is such that if a MR is obstructed by either a lengthy or concave shaped obstacle, the VFF, VOC and VGC should be triggered otherwise implement VFF and VGC else, implement the VFF procedure only. Olunloyo and Ayomoh [47] proposed the HVFF approach to take care of PP in both static and dynamic obstacles scenario [48,49,50,51]. The methodology endeavours to impersonate human knowledge by recognising a nearby local minimal trap causative obstacle as an entity, while continuing away from the trap towards the target point. Despite advances with this technique, there is still limited investigation on this algorithm in environments that can impact on the functionability of sensors. Moreover, an outlook where magnetic field forces can interfere negatively with this algorithm has not been explored especially in underground mines, as the APF group of methods are directly linked to attraction and repulsion of forces from the workspace objects.

#### 2.1.2. Heuristic Approaches

This section presents a review of the following techniques considering their deployment in a VO navigation environment: Fuzzy Logic (FL), Neural Network (NN), Particle Swarm Optimisation (PSO), Genetic Algorithm (GA), Ant Colony Optimisation (ACO), and Firefly Algorithm (FA). In [52,53,54,55,56,57,58,59,60,61,62,63,64,65,66,67,68,69,70,71,72,73,74,75,76,77,78,79,80,81,82,83,84,85,86,87,88,89,90,91,92,93], these algorithms were effectively deployed for RN in presence of DOs and SOs; however, they never experimented for environments, as discussed in Section 1.

##### Fuzzy Logic (FL)

FL was introduced by Zadeh [52] and extensively used in robotics engineering to guide robots. FL control is appropriate for minimal effort robots that do not need highly complex routes and are motivated by human thinking. The behaviour-based FL by Qing-yong et al. [53] includes OA. Jaradat et al. [54] investigated RN in a dynamic environment where they integrated FL with APF. The disadvantage is the LMT, where the robot becomes caught while sitting tight for an obstacle. Pandey et al. [55] developed an FL for taking care of the PP issue in the presence of various states of SOs to discover crash freeway. Outcomes showed that the technique empowers the MR to arrive at the objective without impacting. In the future, an improvement will be by streamlining with the assistance of optimisation algorithms. In [56,57], improvements have been made on FL, but the experiments do not consider environments with VOs, but rather only DOs and SOs. Batti et al. [58] extended the use of FL for OA in labyrinth workspace. Similarly, Mohanty et al. [59] proposed a new model called Takagi-Sugeno FL developed to address PP via an enhanced wall following approach. However, in their paper, the approach was limited to the static obstacles problem. Moreover, in [60] an improvement of FL was proposed by applying it in a complex environment that involves more than two DOs. Moreover, in discussions of the papers reviewed for FL, there are insufficient details on the application of this method in underground mines. The capability of FL in MR is still limited to environments that do not consider toxic environments for sensors (i.e., VOs). An exploration of the effectiveness of this algorithm in different environments remains to be investigated.

##### Neural Network (NN)

NN is a considerable plan of equivalent spread planning segments related to graph geology. It has filled quickly in recognising objects and obstacle discovery in a picture. Recently, the issue of recognising obstacles in the RN system has been essential [61]. The popular methodology used to solve this problem in the past years has been convolutional neural networks (CNN) [62,63]. Chi and Lee [64] used various principles that were actualised for the control technique to keep away from the obstacle effectively. The proposed framework with the NN control approach has illustrated the adequacy of dodging the obstacles. It needs further exploratory examination in other environments with VOs such as underground mining. Moreover, in [65,66,67,68], researchers continued to explore the NN technique with environments consisting of DOs and SOs. Wei and Ye [69] proposed an obstacle avoidance (OA) framework dependent on GA-supported OIF-Elman NN. In their paper, they showed three layers that make up the design of an OIF-Elman network structure. The layers include the input, hidden and output layers. A context layer is also included in the hidden layer. The context layer inside the hidden layer is the primary feedback mechanism of Elman The framework presents a versatile navigation procedure for robots to evade impediments in a workspace. Results presented, showed that the OIF-Elman network is quite successful with OA. This approach was applied in an indoor environment in order to avoid the effect of illumination. Zhang et al. [70] focused on improving NN for RN in complex environments. The simulation results showed considerable efficiency and effectiveness despite the change of different conditions such as weather conditions and road changes. However, the application is not tested in extreme weather conditions where sensors can malfunction.

##### Particle Swarm Optimisation (PSO)

PSO is broadly utilised in versatile RRs tending to RR planning and confinement issues in the obscure workplace [71]. Examination of different methodologies and results showed that the FL matched with PSO provides the ideal outcomes in separation voyages [72]. Atyabi et al. [73] introduced an extension of the PSO in robotics to improve performance. The research considered the environment with SOs and DOs. Results showed potential under the conditions considered; however, the method cannot work fully in robotics. This is very limiting to further investigation for environments with VOs, as they can negatively impact sensors. Future work will examine the effectiveness of this method under real world applications. Another technique named the PSO-IAC [74] is used to determine the objective of approaching the OA issue for a 6° of freedom controller of the home assistance robot. Simulation outcomes demonstrated that the PSO-IAC calculation gives the quickest combination capacity. The suggested control plan can cause the controller of the home assistance robot to show up at the objective situation with and without impediments. However, home environments do not consist mainly of VOs, as it is an environment that is safe for humans compared to underground mining environment.

Meerza et al. [75] built up a PSO-based robot PP calculation that impacts shirking capacity for SOs and DOs. They will test their calculation in a certifiable workplace in the future. Alaliyat et al. [76] proposed powerful PP calculations dependent on PSO to manage the complex dynamic workplace. Outcomes indicated that without any earlier information about a workplace, the robot could accomplish its objective of evading SOs and DOs. In the future, they plan to acquire a super robot that can learn and retain the circumstances during its navigation. It’s not clear if VOs would form part of the future consideration for their proposed real experiment. Tian et al. [77] deployed the use of remote sensing in finding multiple robots and impediments, which utilised an improved counterfeit clever calculation. One limitation to the method is the calculation of the union speed to improve the worldwide pursuit execution and failure to manage the circumstance that numerous robots may collide. In the future, a hypothetical exploration of PSO calculation and obstruction evasion calculation to manage different testing improvement issues will be studied. The noticeable gap in discussion of PSO means that there are insufficient details when it comes to its effectiveness in environments with obstacles that makes sensors malfunction. Underground mining is an environments where this technique still needs to be explored for effectiveness and efficacy.

##### Genetic Algorithm (GA)

GA is a known technique-based enhancement instrument that follows the guideline of hereditary qualities and joint determination [78]. Application of this technique to software engineering was introduced first in 1975 [79]. The utilisation of GA for the versatile RR issue is significant in the static workplace. Reproduction results introduce the investigation as they were within sight of a polygonal impediment. Xiao et al. [80] embraced the strategy to accomplish the objective of the route. Many scientists have given less attention to the sight of a DO in an uncertain workplace [81]. To improve results in robot PP, numerous scientists have joined in on using GA and other techniques to obtain a HA [7]. Patle et al. [82] state that in the future, the work may stretch to cause the crossbreed regulator for the ongoing open-air workplace usage. Germi et al. [83] tended to alter the first potential field calculation to better the exhibition of the calculation in dynamic conditions. Choueiry et al. [84] introduced a survey of the PP enhancement issue and a calculation for robot PP in a static environment using GA as a device to discover number of steps while staying away from obstacles. The designed flowchart of the proposed approach took into consideration, the workspace grid size, initial and target positions and obstacles distribution all serving as inputs. If the MR does not reach the TP the number of steps in the GA algorithm are increased. Lopez-Gonzalez et al. [85] utilised GA to accomplish distance-based development by using two unique sorts of chromosomes. Aghda and Mirfakhrae [86] consolidated the GA-FL to improve directing. In the quest to improve GA, this approach is incompetent in dynamic environments [50], but produces good outcomes in simulation. However, as this approach has not been tested in environments with VOs, this is open for future investigation.

##### Ant Colony Optimisation (ACO)

ACO applies in the robot system field, particularly the PP issue [87]. ACO resolves this issue to determine the mechanical flying-vehicle course for a war zone [88]. Zhangqi et al. [89] proposed improvement measures and applied GA to the advancement and arrangement boundaries of the essential ACO. Simulation outcomes showed that the improved ideal path length is essentially not exactly the fundamental ACO. Wang et al. [90] improved APF first, implementing a strategy for a piecewise capacity of fascination potential that suggests that the robot can, without much of a stretch, slam into the obstruction. The limitation of this approach is that the model contains numerous boundaries which makes it difficult to tune. Even the flowchart design depicts this limitation by having a lot of decision blocks. Researchers will discover the relationships between these boundaries in the future. Yi et al. [91] produced dynamic change data as indicated by the contrast between the best way of the past age and the current ACO cycle. Ma et al. [92] addressed the automated submerged vehicle two-dimensional independent PP issue in the climate influenced by sea momentum and obstacles. Results showed that this calculation could rapidly locate the ideal global arrangement where the unpredictable workplace is. However, there is no clear indication on the impact of these conditions on the sensors. Zhao [93] proposed the ideal way of anticipating whether robots are dependent on ACO by contemplating the connected writing and effective methods of robot PP. Results showed that the model could wisely pick a robot with DO evasion efficiently.

## 3. Discussion

It is clear that there is a noticeable gap in the literature in respect of VOs, as there is insufficient consideration, information, or discussions about such environments where sensors can fail, or inefficacy of an algorithmic output based on environmental influences on mounted sensors. The researchers very often present discussions on the efficiency of the deployed algorithms without referring to sensory incapacitation even in a possible medium- to high-risk experimental environment. Mostly, these algorithms were validated on simulation platforms with a few validated experimentally using real dynamic obstacles (DOs) and/or real static obstacles (SOs) environment.

In as much as some measures appear to be in place regarding the combating of VOs in both open and obscured environments for MRs, for instance LiDAR generally uses various filtering methods to filter dust while real industrial robots generally have redundant sensors to process information to ensure their stable operation under VO conditions etc., this paper recommends a concept premised on holistic path planning. Holistic path planning should integrate VOs thinking as much as real obstacles thinking in robot navigation problems and solution proffering. While purposeful experiments on robot navigation to examine the efficacy of general sensorial incapacitation due to extreme or obscured environmental factors are still lacking in the literature (see introductory sections), future research will present robot vehicle navigation limitations based on sensorial incapacitated experiments. The experiments will utilise the same set of robotic vehicles in two different navigation environments depicting different (i.e., normal and extreme) environments over different trials with the conduct of statistical significance of the difference in navigation output over time. In addition, it is recommended that in a traditional robot navigation task, when obstacle avoidance and goal reachability becomes challenging, a robotist should verify the functionality of the onboard sensors, power unit, and actuators. If all are in a good operational condition, VOs capable of incapacitating the onboard sensor types may likely be in play and should be verified. This troubleshooting recipe can be of a greater assistance in extreme or obscured environments involving robot vehicle navigation.

Furthermore, following that VOs can interfere with or influence both the internal and external workings of nearly all sensor devices through the interception of both receptive and emitted sensorial signals, leading to wrong computation and misleading robot navigation decision, an additional measure of solution to address a possible external influence can revolve around the integration of a machine learning assisted algorithm for sensors response accuracy and interpretation of propagated signals. Based on this proposed solution, each time a sensor emits and receives a feedback signal from the external environment based on the obstacles along its navigation path, the machine learning algorithm should be able to compare the most recent and similar emitted signal from its historic emissions and see if the disparities between the receptive signals for the same or similar emitted signals are significantly different. In the case of a significant difference, the robot can send out a beep sound, which is an indication of a possible external interference to its sensorial computation. However, regarding internal distortions caused by VOs, a sensor-proof capability, which would protect the limitations as explained in previous sections, can go a long way in securing the hardware.

### Key Findings from This Research Are as Presented Below

Few papers have addressed the RN problem in the presence of VOs. Virtual obstacles are not visible to both the mounted sensors and the human eyes. However, these can affect the navigation of a robot towards the desired TP by interfering with the operations of the sensors, resulting in wrong sensorial output metrics. Examples of experimentally unverified VOs include magnetic field influence on sensors (infrared sensors), extreme temperature effect on sensors (freezing temperature, boiling temperature), as well as infrared sensors, electric field effect on sensors, and so forth.

Occasionally, the effect of frictionless navigation environment on the navigating wheels of a robot can also impede the display of intelligence in target point attainment. Even though frictionlessness is not a VO in the context of an obstruction, it serves as a virtual impedance to a MR in its bid to accurately reach and stop at a desired target point. Also, in a noisy, clustered environment, the performance of a sonar sensor can be subject to some form of impedance. Furthermore, a vision system-controlled navigation can be influenced by the degree of illumination a robot is exposed to.

Furthermore, additional significant limitations with some of the methodologies presented in the literature is the processing speed and performance in complex nonconventional navigation environments as a result of certain environmental impediments. Future work in this field will present specific sensory experimental quantitative information covering different VO prone environments as earlier presented.

## 4. Conclusions

This paper has explored the problem of VOs in robot navigation obstruction in certain extreme or obscured navigation environments as a robot travels from one point to another within the workspace. Based on this, the current review has investigated robot navigation incapacitation resulting from environmental conditions that can hamper the performance of a sensor. The review is premised on the fact that sensory incapacitation, just as with algorithmic ineffectiveness, can hinder a robot from successfully navigating to a desired position in a given workspace. Sensory incapacitators in the context of this review are not orchestrated by visible, physical objects, but rather by invisible, virtual phenomena as earlier presented. Furthermore, based on the possible influence of VOs on the navigation intelligence of robots due to sensory incapacitation, the robust and all-encompassing concept of SLAM, as previously discussed in this review, is considered to be more skewed towards algorithm effectiveness in the control of a robot than the tracking of a robot’s hardware incapacitation, nevertheless with a generic consideration given to onboard hardware units. It is quite obvious that there are not any categoric considerations for sensors incapacitation based on VOs (see Durrant-Whyte and Bailey [17]; Taheri and Xia [13]). Based on the above, it is suggested that the broad concept of SLAM be extended or modified to address both “algorithm effectiveness and sensors signal” (emission and reception) monitoring and evaluation, especially when a robot is navigating in an obscured environment. This can be achieved by deploying a modified concept of SLAM with the acronym “SLAAAM”, representing “Simultaneous Localisation Assessment Adaptation and Mapping”. The assessment component in “SLAAAM” which is the first “A”, would address the disruption in sensory signal emission and reception and prepare the robot for “adaptation” which is the second “A”. The assessment would be carried out by way of a swift analysis and evaluation of receptive signals. The deployment of the assessment process will require an onboard vision sensor with both (obstacle proximity response measurement and imaging capability) and a non-vision sensor with (obstacle proximity response measurement capability).

Usually in an operational environment, a vision sensor will scan the ambient environment to generate images of physical obstacles while also keeping record of the measured obstacle’s distance during the simultaneous mapping process. Similarly, the non-vision sensors (e.g., infrared or sonar sensors) would intermittently send out and receive sensory signals for proximity distance measurement from obstacles in the ambient environment. This assessment process is such that when the processed receptive signal by the vision and non-vision sensors are somewhat misaligned, not necessarily with each other but with their default sensing attributes when they sense obstacles (for instance, a vision sensor capturing no obstacle image yet exhibiting some sensory receptive features in response to a non-existent obstacle), may arguably signify the presence of a sensory interceptive medium which is obviously a virtual obstructive medium.

Even though different sensors are expected to react to different VOs based on their operational mode, their respective incapacitative response would remain the same for every VO they are prone to. For instance, a vision sensor will often not be able to produce any captured image when a virtual rather than a physical obstacle is present within its sensing zone. However, the non-vision sensors such as infrared and sonar will have their emissions intercepted and a false receptive response signal propagated. Finally, the adaptation component of “SLAAAM” would prompt the robot to respond to an unusual obstacle scenario as depicted by the assessment process above, hence causing the affected sensing devices to be triggered off intermittently as the robot withdraws from the affected sensory incapacitated mapped region to avoid a partial or absolute damage of the incapacitated sensing device. The sensors are left in the normally “on” status and immediately the robot is out of the mapped incapacitated region.

Table 1 presents a summary of pathplanning methodologies discussed above and their taxonomy covering: Types of obstacles, obstacle geometry, approach used, results, year, TP and number of robot(s) deployed. Additionally, the taxonomy breakdown covers references where the algorithms were tested for effectiveness and deployment ommited the ones used just for the literature.

## Figures and Tables

**Table 1:** Analysis of various path planning and navigation algorithms amidst obstacles.

Ref No	Techniques	Environment Consists of			Technique Used as		Result		Year	Obstacle(s) Shape		Target Point (TP)		Robot	
		SOs	DOs	VOs	Stand Alone	Hybrid	SR	RTR		Cv	Cx	Single TP	Multi TP	Single Robot	Multi Robot
Classical Approach															
[12]	SLAM	Yes	No	Yes	Yes	No	No	Yes	2019	Yes	Yes	Yes	No	Yes	No
[13]		No	No	No	Yes	No	No	No	2021	No	No	No	No	No	No
[14]		Yes	No	No	Yes	No	No	Yes	2014	Yes	Yes	Yes	No	Yes	No
[15]		No	Yes	No	No	Yes	Yes	No	2014	No	Yes	Yes	No	Yes	No
[16]		Yes	Yes	No	Yes	No	No	Yes	2016	Yes	Yes	Yes	No	Yes	No
[17]		Yes	No	No	Yes	No	Yes	Yes	2006	Yes	Yes	Yes	No	Yes	No
[18]		Yes	Yes	No	No	Yes	No	Yes	2018	Yes	Yes	Yes	No	Yes	No
[19]		Yes	Yes	No	No	Yes	Yes	No	2021	Yes	Yes	Yes	No	Yes	No
[20]		Yes	No	No	Yes	No	Yes	Yes	2021	No	Yes	Yes	No	Yes	No
[21]		Yes	No	No	Yes	No	Yes	No	2021	No	Yes	No	No	Yes	No
[23]	Light Detection and Ranging (LiDAR)	Yes	No	No	Yes	No	Yes	No	2017	No	Yes	Yes	No	Yes	No
[24]		Yes	No	No	Yes	No	Yes	No	2019	No	Yes	Yes	No	Yes	No
[25]		Yes	No	No	Yes	No	No	Yes	2019	Yes	Yes	Yes	No	Yes	No
[26]		Yes	Yes	No	Yes	No	Yes	Yes	2020	Yes	Yes	Yes	No	Yes	No
[27]		Yes	Yes	No	Yes	Yes	No	Yes	2020	Yes	Yes	Yes	No	Yes	No
[29]	Vector Field Histogram (VFH)	Yes	No	No	Yes	No	No	Yes	1991	No	Yes	Yes	No	Yes	No
[30]		Yes	No	No	Yes	No	No	Yes	1998	No	Yes	Yes	No	Yes	No
[31]		Yes	No	No	Yes	No	Yes	Yes	2000	No	Yes	Yes	No	Yes	No
[32]		No	Yes	No	Yes	No	No	Yes	2012	No	Yes	Yes	No	Yes	No
[33]		Yes	No	No	Yes	No	Yes	No	2014	No	Yes	Yes	No	Yes	No
[34]		Yes	Yes	No	Yes	No	No	Yes	2016	Yes	Yes	Yes	No	Yes	No
[35]		Yes	Yes	No	Yes	No	Yes	No	2019	No	Yes	Yes	No	Yes	No
[36]		Yes	Yes	No	Yes	No	No	Yes	2020	No	Yes	Yes	No	No	Yes
[37]	Artificial Potential Field (APF)/ Virtual Force Field (VFF)	Yes	No	No	Yes	No	Yes	No	2014	No	Yes	Yes	No	Yes	No
[38]		Yes	No	No	Yes	No	No	Yes	1989	Yes	No	Yes	No	Yes	No
[39]		Yes	No	No	Yes	No	No	Yes	1985	Yes	Yes	Yes	No	Yes	No
[40]		Yes	Yes	No	No	Yes	Yes	No	2015	Yes	Yes	Yes	No	Yes	No
[41]		No	Yes	No	No	Yes	Yes	No	2017	Yes	Yes	Yes	No	Yes	No
[42]		Yes	No	No	Yes	No	Yes	No	2018	Yes	No	Yes	No	Yes	No
[43]		Yes	No	No	Yes	No	Yes	No	2020	No	Yes	Yes	No	Yes	No
[44]		Yes	No	No	Yes	No	Yes	No	2020	Yes	Yes	Yes	No	Yes	No
[45]		Yes	Yes	No	Yes	No	Yes	Yes	2019	No	Yes	Yes	No	Yes	No
[46]		Yes	No	No	No	Yes	Yes	Yes	2021	Yes	Yes	Yes	No	Yes	No
[49]		Yes	No	Yes	No	Yes	Yes	No	2009	Yes	Yes	Yes	No	Yes	No
[48]		No	Yes	Yes	No	Yes	Yes	No	2009	Yes	Yes	Yes	No	Yes	No
[49]		No	Yes	No	No	Yes	Yes	No	2010	Yes	Yes	Yes	No	Yes	No
[50]		Yes	No	No	No	Yes	Yes	No	2011	Yes	Yes	Yes	No	Yes	No
[51]		Yes	No	No	No	Yes	Yes	No	2021	Yes	Yes	Yes	Yes	Yes	No
Heuristic Approach															
[53]	Fuzzy Logic (FL)	Yes	No	No	Yes	No	Yes	No	2009	Yes	Yes	Yes	No	Yes	No
[54]		Yes	Yes	No	No	Yes	Yes	No	2012	No	Yes	Yes	No	Yes	No
[55]		Yes	Yes	No	Yes	No	Yes	No	2014	No	Yes	Yes	No	Yes	No
[56]		Yes	Yes	No	Yes	No	Yes	Yes	2016	Yes	Yes	Yes	No	No	Yes
[57]		Yes	No	No	Yes	No	Yes	Yes	2018	No	Yes	Yes	No	Yes	No
[58]		Yes	No	No	Yes	No	Yes	No	2019	Yes	Yes	Yes	No	Yes	No
[59]		Yes	No	No	Yes	No	Yes	Yes	2020	Yes	Yes	Yes	No	Yes	No
[60]		Yes	Yes	No	Yes	No	Yes	No	2021	No	Yes	Yes	Yes	Yes	Yes
[64]	Neural Network (NN)	Yes	No	No	Yes	No	No	Yes	2011	No	Yes	Yes	No	Yes	No
[65]		Yes	No	No	Yes	No	Yes	No	2014	No	Yes	Yes	No	Yes	No
[66]		Yes	No	No	Yes	No	Yes	No	2004	Yes	Yes	Yes	No	Yes	No
[67]		Yes	Yes	No	Yes	No	Yes	No	2019	No	Yes	Yes	No	No	Yes
[68]		Yes	No	No	Yes	No	No	Yes	2020	Yes	Yes	Yes	No	Yes	No
[69]		Yes	No	No	No	Yes	Yes	No	2020	Yes	Yes	Yes	No	Yes	No
[70]		Yes	Yes	No	Yes	No	Yes	No	2020	Yes	Yes	Yes	No	Yes	No
[73]	Particle Swarm Optimisation (PSO)	Yes	Yes	No	Yes	No	Yes	No	2010	No	Yes	Yes	No	Yes	No
[74]		Yes	Yes	No	Yes	No	Yes	No	2016	No	Yes	Yes	No	Yes	No
[75]		Yes	Yes	No	Yes	No	Yes	No	2018	Yes	Yes	Yes	No	Yes	No
[76]		Yes	Yes	No	Yes	No	Yes	No	2019	Yes	Yes	Yes	No	Yes	No
[77]		Yes	Yes	No	Yes	No	Yes	No	2021	Yes	Yes	No	Yes	No	Yes
[83]	Genetic Algorithm (GA)	Yes	Yes	No	Yes	No	Yes	Yes	2018	No	Yes	Yes	No	Yes	No
[84]		Yes	No	No	Yes	No	Yes	No	2019	Yes	Yes	Yes	No	Yes	No
[85]		No	Yes	No	Yes	No	Yes	Yes	2020	No	Yes	No	Yes	No	Yes
[86]		Yes	Yes	No	No	Yes	Yes	No	2020	No	Yes	Yes	No	Yes	No
[89]	Ant Colony Optimisation (ACO	Yes	No	No	Yes	No	Yes	No	2011	Yes	Yes	Yes	No	Yes	No
[90]		Yes	No	No	No	Yes	Yes	No	2018	Yes	Yes	Yes	No	Yes	No
[91]		Yes	No	No	Yes	No	Yes	No	2019	Yes	Yes	Yes	No	Yes	No
[92]		Yes	Yes	No	No	Yes	Yes	No	2020	No	Yes	Yes	No	Yes	No
[93]		Yes	Yes	No	Yes	No	Yes	No	2020	Yes	Yes	Yes	No	Yes	No

Cv = Concave, Cx = Convex, SR = Simulation Result, RTR = Real Time Result.

## Data Availability

Not applicable.
